# A narrative review on the burden of migraine: when the burden is the impact on people’s life

**DOI:** 10.1186/s10194-019-0993-0

**Published:** 2019-04-25

**Authors:** Matilde Leonardi, Alberto Raggi

**Affiliations:** Neurology, Public Health and Disability Unit, Fondazione IRCSS Istituto Neurologico Carlo Besta, Via Celoria 11, 20133 Milan, Italy

**Keywords:** Migraine, Burden, Disability, Quality of life, Years lived with a disability, Disability-adjusted life years, Work impact, Family impact, Interictal, Cost

## Abstract

**Background:**

The burden of headache disorders, and of migraine in particular, is multifaceted and fragmented. The aim of this narrative review is to provide a description the main topics underlying the concepts of burden and impact of migraine disorders.

**Main results:**

MedLine has been searched for publications covering the period 1990–2018 dealing with the terms burden or impact of migraine, including both episodic and chronic migraine. The main results and themes are reported in a descriptive way, and were grouped by similarity of content into overarching categories. A total of 49 papers, published over 25 years (1994–2018), were retained for the qualitative analysis. Six main themes were identified: prevalence of migraine disorders, overall impact of migraine disorders, impact on work or school activities, family impact, interictal burden, and disease costs. Majority of included studies concluded that patients with migraine reported an higher burden or impact in one or more of the six main themes herein identified, compared to non-headache patients or to patients with tension-type headache, with a tendency towards worse outcomes consistently with higher headache frequency.

**Conclusions:**

The results of this narrative review show that the meaning of a sentence like “migraine is a burdensome condition” is not univocal: rather, it may refer to different concepts and meanings. In our opinion, future research should focus on understanding and facing the impact of migraine on work-related activities and on everyday life activities, as these aspects are highly connected to some tangible (i.e. cost) and less tangible (i.e. interictal burden and reduced quality of life) facets of migraine burden. Disease-specific measures have been implemented and should be exploited to enhance our understanding of migraine burden. This approach would allow to better understand the real impact on people’s life of such a burdensome disease.

## Introduction

The recent report from the Global Burden of Disease (GBD) 2015 Neurological Disorders Collaborator Group shows that a broad group of neurological disorders – i.e. a set of disease categories in which stroke, brain cancers, tetanus, encephalitis and meningitis were added to the usual set of neurological disorders – accounts for 250.7 million Disability-Adjusted Life Years (DALYs), i.e. 10.2% of global DALYs, and it increased by 7.4% in the last 25 years [1]. Based on Institute for Health Metrics and Evaluation’s data, the same neurological disorders account for 85.6 million Years Lived with a Disability (YLDs) in 2015, i.e. 10.7% of global YLDs, that however increased by 55.2% (YLDs were 55.1 million in 1990) in the last 25 years [2]. The last 25 years, i.e. since the first publication of GBD-1990 in 1994 [3], enabled researchers to get to a core point in health policy making: being counted to count.

A considerable portion of the burden of neurological disease is due to headache disorders: data from GBD 2015 in fact show that tension-type headache (TTH) and migraine are the most common conditions and they account for 60.3% of YLD associated to brain conditions (respectively, 7.2 and 44.5 million YLDs in 2015). The considerable burden associated to migraine and TTH is connected to the huge prevalence of these conditions: in absolute terms, it has been increasing in the last decades, but when addressed in terms of age-standardized rates, it seems to be basically stable or moderately declining [4]. The same happens when YLDs are taken into account: they increased in absolute terms, but are basically stable in terms of age-standardized rates.

Taken as a whole, these data suggest that reducing the burden of headache disorders, and of migraine in particular, should be a primary objective for health policymakers: however, what should policies aim to, for reducing burden of migraine, is still a matter of debate. In fact, dealing with the “natural” within-person variation in migraine headache frequency, which is the fundamental driver of the instability in diagnostic classification for episodic and chronic migraine (EM and CM) [5–8] is inevitable. As a consequence, addressing the impact of migraine disorders is made difficult by the intrinsic nature of migraine headaches, and by issues connected to the daily tasks patients carry out and that can be to different extents impaired by migraine headaches: patients may in fact be limited in their daily life functioning during ictal phases, and able to perform daily duties with higher capacity during interictal ones (although the presence of an interictal burden in migraine has been shown [9, 10]). The debate on which domains or daily life are mostly affected by migraine headaches is therefore not easy to address and needs further studies. The most used disability measure in migraine, the Migraine Disability Assessment (MIDAS), includes questions on work, homework and leisure time [11]. However, each of these macro-areas – and the work-related ones in particular – are constituted by several sub-components dealing with specific activities connected to body movements (e.g. lifting objects, walking or driving) as well as skills connected to communication and entering into relation with others [12–14]. These difficulties clearly depend on migraine features, in terms of frequency as well as of symptoms severity, but are also highly dependent on several person-level features, e.g. patients’ lifestyle, and on the features of their jobs and of the environment in which they live. Furthermore it cannot be forgotten that the burden of headache disorders, and of migraine in particular, is also an economic burden, where work-related costs also due to absenteeism, presenteeism and reduced productivity constitutes most of the economic impact [15–19].

It can be therefore concluded that the burden of migraine is multifaceted and the lack of clear information on the different aspects of migraine burden may produce fragmented research results: this, in turn, may hinder the identification of intervention target and, as a consequence, limits the effectiveness of public health policies. In fact, on one side, patients enrolled in different studies are generally asked questions on few domains, such as those included in MIDAS: therefore our notion on the impact of migraine from the patients’ perspective is limited to few domains. Parallel to this, studies strictly addressing migraine burden rely on YLDs and DALYs, and eventually on direct and – more rarely – on indirect disease’s costs: in these cases the perspective is not patient-centred, but society-centred. To the best of our knowledge, no study has addressed the question “what is practically behind the concept of burden” and, to enhance our ability to identify future research and policy targets, we need to understand what for migraine is behind the “D” of YLDs and DALYs. The aim of this paper is therefore to provide a response to such a question by addressing the existing literature jointly dealing with the impact and burden of migraine disorders. To pursue this objective, we performed a revision of the literature, relying on a narrative approach, to describe the main topics underlying the concept of burden and impact of migraine.

## Methods

We revised scientific literature published since 1990 onwards, and searched for publications dealing with impact and burden in episodic and chronic migraine by searching on MedLine. We selected 1990 as start of search because the first GBD study was referred to 1990, and the beginning of the 90s is the period in which the World Bank commissioned the first GBD study [3]. We searched in manuscript’s titles and abstracts for the term “migraine” and the terms “impact AND burden”. We went through abstracts and excluded records not dealing with the topic or with EM or CM, dealing with mixed populations (e.g. studies addressing the burden due to migraine comorbidity in patients with other conditions, or studies addressing burden of headache disorders in general), not in English or without abstract. When we moved to full-text analysis we excluded studies that were out of topic, that did not report extractable data (e.g. commentaries, editorial or conceptual papers, papers reporting data in a poor-quality way or papers reporting prevalence-based estimates) and studies on populations with mixed headache disorders: in the case of mixed studies, however, we retained papers when data were presented separately, so that they could be clearly referred to EM or CM.

We relied on a narrative approach to analyze and report our results. Therefore, rather than relying on pre-defined concepts, we addressed the main results and themes covered by selected manuscript in a descriptive way and grouped them into overarching categories by similarity of content according to Popay’s guidelines on how to analyze narrative reviews [20]. Once these main themes were identified, we addressed the trend over time of each theme and reported the core results. Therefore, the qualitative synthesis is a summary of the way in which our research question, i.e. the description of the main topics underlying the concepts of burden and impact of migraine, have been addressed in the literature.

## Results

**Fig. 1:**
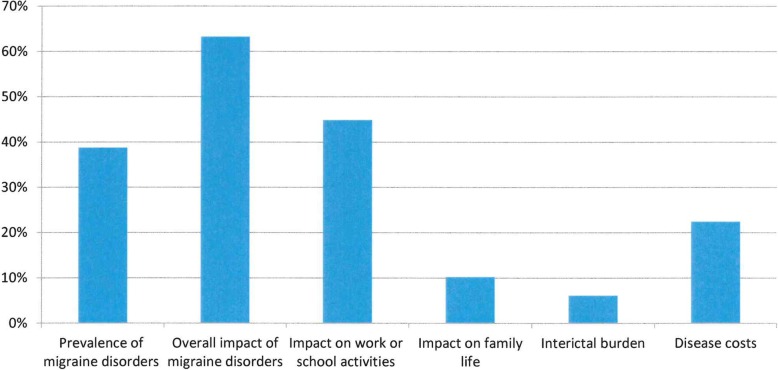
Main themes recurrence across publications

**Fig. 2:**
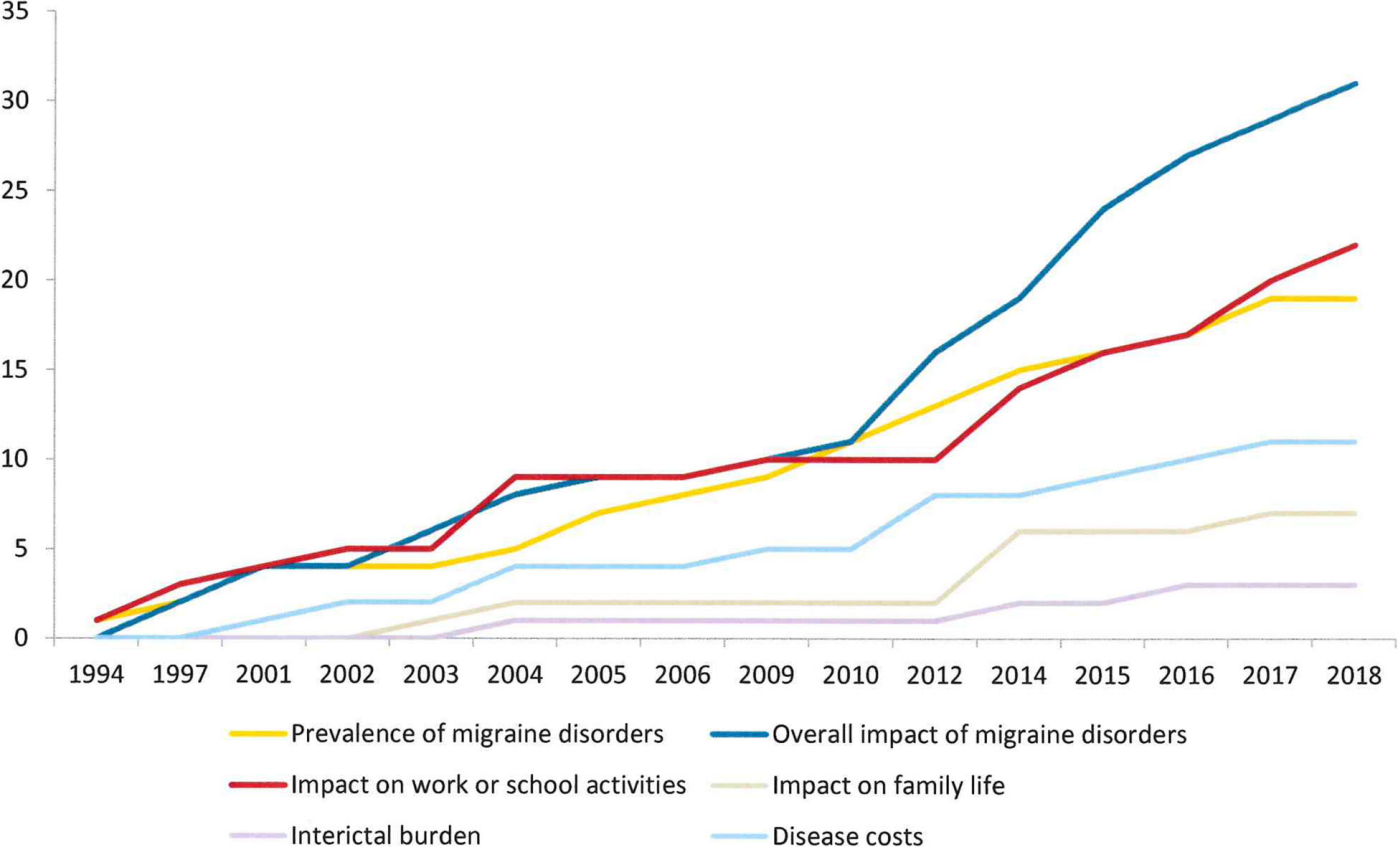
Trend over time for main selected themes

Our initial search retrieved 154 records, of whom 49 were retained for the qualitative analysis [9, 13, 14, 17, 21–65] and were published over 25 years (1994–2018). Six main themes were identified: prevalence of migraine disorders; overall impact of migraine disorders; impact on work or school activities; impact on family life; interictal burden; disease costs. Table 1 reports the main results from selected papers and the main themes across them, Fig. 1 shows the percentage of themes’ recurrence across publications and Fig. 2 shows the trend over time for these themes using cumulative frequencies: this enables to appreciate the presence of a consistent growth gradient for the themes “overall impact of migraine disorders” and “impact on work or school activities” since 2010 onwards.

**Table 1:** Main results and main themes of selected publications

Reference	Country	Sample size	Main themes						Study’s main results
			Prevalence	Overall impact	Work/school impact	Family impact	Interictal burden	Cost	
Raggi 2018 [39]	Italy	373		√	√				A new questionnaire to address migraine impact on work-related activities is presented.
Al-Hashel 2017 [21]	Kuwait	3588	√	√	√				Prevalence of EM in Kuwait is 23.1%; patients lost 2 workdays/3 M and further 4.2 of household and leisure activities
D’Amico 2018 [40]	Italy	194		√	√				CM patients with 2+ comorbidities showed lower QoL and higher disability compared to those with one or less comorbidities, and were more likely to be unemployed (73.7% vs 25.8%)
Rastenytė 2017 [22]	Lithuania	137	√	√	√				Prevalence of EM in Lithuania was 18.8%, for pMOH 3.2%; patients lost 2.8 workdays/3 M and further 6.5 of household and leisure activities
Lipton 2017 [63]	US	13,064				√			A new questionnaire to address migraine impact on partners and adolescent children is presented.
D’Amico 2017 [59]	Italy	135			√			√	CM patients at the time of withdrawal lost 22.3 workdays/3 M; one-year CM cost was estimated at 10730€, and 61% of that cost was indirect
Steiner 2016 [23]	India	615	√	√					Prevalence of EM in Karnataka was 25.2%, for pMOH 1.2%; the day before, 14% of patients lost all productive time, and 47% lost more than half
Lampl 2016 [9]	EU countries	3208					√		Interictal burden was reported by 10–26% of EM patients and by 29–41% of pMOH.
Messali 2016 [41]	US	1205		√	√			√	Patients lost 0.9 workdays/3 M and further 8.4 of household activities. Total cost of EM was 2649$/year, of CM 8243$/year; 60–64% of cost was due to direct medical costs
Raggi 2016 [42]	Italy	80		√					A new questionnaire to address psycho-social difficulties in brain disorders is used in EM patients for the first time; higher levels of PSD were predicted by younger age, higher migraine frequency, higher comorbidities index, and smoking status
Manandhar 2015 [43]	Nepal	774		√	√				Patients lost 2.3 workdays/3 M and further 11.4 of household and leisure activities; EM and pMOH patients had worse QoL compared to non-headache patients.
Berra 2015 [44]	Italy	92		√				√	Patients lost 8,8 workdays/3 M and further 12.9 of household activities. Total direct healthcare cost of EM was 521€/year, of CM 2250€/year.
Pavlović 2015 [45]	US	1697		√					Patients with migraine related to menses reported higher disability and disease impact
Queiroz 2015 [24]	Brazil	2345	√						Prevalence of EM in Brazil is 15.8%,of pMOH is 6.1%; patients with migraine and pMOH showed higher disability compared to TTH.
Raggi 2015 [46]	Italy	194		√					CM patients report higher disability compared to normative scores; patients with higher severity report worse QoL and disability; patients lost 6 workdays/3 M and further 20 in homework and leisure activities.
D’Amico 2015 [47]	Italy	296		√	√				EM and CM have a relevant impact on work-related difficulties, and patients reporting higher difficulties in work-related tasks also show problems in tasks unrelated to work.
Wöber-Bingöl 2014 [48]	Austria	472		√	√	√			44.9% of pupils reduced or missed school-days; QoL was worse consistently with headache frequency; parents had to reduce workforce participation to care for children during attacks.
Steiner et al. 2014 [17]	EU countries	2109	√	√	√	√	√		Prevalence of EM in EU is 22.2%, of pMOH is 3.3%; patients lost 4.6 workdays/3 M and further 9.8 days of household and leisure activities; overall impact, interictal burden and family burden increased consistently with increased headache frequency.
Ayzenberg et al. 2014 [25]	Russia	411	√	√	√				Prevalence of EM in Russia is 20.3%; patients lost 0.2 workdays/3 M and further 2 days of household and leisure activities; patients with EM showed higher impact compared to those with TTH and higher indirect costs.
Raggi 2014 [14]	Global	51,135			√				Specific difficulties in work-related tasks are poorly addressed and are confined to few activities, the most common being speaking and driving
Raggi 2012 [13]	Global	20,852		√					EM has a pervasive impact on several life domains, which is influenced by pain severity and headache frequency
Bloudek 2012 [49]	EU countries	5657		√				√	Patients with CM reported higher disability compared to those with EM; the average direct cost of EM was 746€/year, that of CM was 2427€/year
Manhalter 2012 [50]	Hungary	168		√					Patients with EM had lower QoL compared to those with TTH
Yu 2012 [26]	China	5041	√	√				√	Prevalence of EM in China was 9.3%, of pMOH was 1.6%; patients with EM and pMOH had worse QoL and higher disability compared to TTH and no-headache; EM and pMOH had higher cost compared to TTH
Silva Junior 2012 [27]	Brazil	47	√						Prevalence of migraine (EM and CM) is 18.2%
Buse 2012 [51]	US	6927		√					CM has higher impact compared to EM
Linde 2012 [66]	EU countries	2844						√	The mean per person annual cost of EM was 1222€, that of MOH was 3561€; indirect cost accounted for more than 90% of total cost
Cooke 2010 [28]	Canada	1210	√						Migraine prevalence among females in Canada is 26%
Stovner 2010 [29]	EU countries	170,000	√						Prevalence of EM is estimated at 14.7% among adults and at 9.2% among children; prevalence of pMOH is estimated at 4% among adults and around 1% among children
Leonardi 2010 [52]	Italy	102		√					EM patients report lower QoL and higher disability compared to normative scores; patients with higher severity report worse QoL and disability; patients lost 6 workdays/3 M and further 20 in homework and leisure activities.
Munakata 2009 [60]	US	7796			√			√	Patients with EM lost 4.7 workdays/year, those with CM 26.7; per-person/year cost of EM was 1757$, of CM was 7750$
Radtke 2009 [30]	Germany	769	√	√					Prevalence of migraine in Germany is 10.6%; compared to other headache sufferers, migraineurs were more likely to report higher disability rates, consume more analgesics and attend medical consultation
Stovner 2006 [31]	Global	5465	√						Prevalence of EM was 14%, of CM 4%
Dueland 2005 [32]	Global	760	√	√					Prevalence of EM was 42% in young women; 86% reported negative impact on daily life activities
Lipton 2005 [33]	Global	18,897	√						Prevalence of EM was 9.2%
Bussone 2004 [53]	Italy	414		√	√				Patients with CM reported higher disability compared to those with EM; patients lost 13.1 workdays/3 M and further 30.3 of household and leisure activities.
Vicente-Herrero 2004 [61]	Spain	436			√			√	After an on-work consultation (acute and prophylactic treatment plus lifestyle-related advices) patients reduced the total workdays lost equivalent from 0.5 days/month to 0.1; total per-migraine headache productivity cost was reduced from 34.5€ to 4.6€.
Pradalier 2004 [34]	France	880	√		√			√	Prevalence of EM in France is 7.9%,of pMOH 3%; EM patients lost 0.5 workdays/3 M and further 0.6 of reduced productivity; total direct cost of EM was 128€/year
MacGregor 2004 [54]	Global	866		√	√	√			Patients lost 5.5 workdays/3 M and further 13.4 days of household and leisure activities; most of partners of patients reported that living with a migraineur has moderate/strong impact on family life and leisure time
Stonks 2004 [65]	The Netherlands	24					√		During inter-ictal periods, compared with healthy controls migraine patients spent relatively less time being active and, when active, their overall body mobility was lower; they also reported higher sleepiness and lower vigour
Ware 2003 [55]	US/UK	221		√					Patients with migraine reported higher HIT-6 scores compared to patients with other headache disorders
Lipton 2003 [56]	US/UK	389		√		√			Approximately half of the patients reported limitations in daily family activities; the majority reported limitations in activities dealing with children.
Edmeads 2002 [62]	US	1079			√			√	Patients missed approximately 50% more workdays compared to controls, attended more outpatient visits and ER access, and reported global disease cost at 1242$/year, 3,4% higher compared to non-migraine controls
Lipton 2001 [35]	Global	10,654	√					√	Prevalence of migraine in the general population across studies was 8.3%, higher in women than in men (between + 7% and + 279%); on average, direct cost was between 100 and 800$ per patient/year
Gerth 2001 [57]	Global	2604		√	√				Patients lost 4.9 workdays/3 M and further 4 days of household activities
Lipton 2001 [36]	US	6915	√	√					Prevalence of migraine in US population is around 12%; more than half of patients reported severe disability/bed rest as impact of migraine
Michel 1997 [58]	France	989		√	√				A total of 49.1% of migraine patients reported health impairment, which was higher than healthy controls; also, patients showed higher anxiety levels and lower QoL. Migraineurs were more likely to report sick leave compared to controls (73% vs. 65.7%) and to lose more than 8 workdays/year (61% vs. 49%). Finally, they reported lower work performance
Solomon et al. 1997 [37]	Global	6794	√	√	√				Prevalence of migraine is 10.7%; migraineurs reported worse QoL scores; patients with migraine lost between 2 and 7 workdays per year
Abu-Arefeh et al. 1994 [38]	UK	159	√		√				Migraine prevalence among school-aged children was 10.6%; children with migraine lost 4.1 schooldays because of migraine

Notes. *EM* Episodic Migraine, *CM* Chronic Migraine, *TTH* Tension-Type Headache, *pMOH* probable Medication Overuse Headache, *QoL* Quality of Life, *EU* European Union, *HIT-6*, six-item Headache Impact Test

### Prevalence of migraine disorders

Information on EM and CM prevalence was reported in 19 studies [17, 21–38]. Based on these studies, prevalence of EM was reported between 7.9% in France [34] and 25.2% in India’s Karnataka State [23], and it peaked up to 42% in a selected sample of young-age women [32]; prevalence of migraine disorders with monthly frequency higher than 15 days (irrespectively of the presence of Medication Overuse Headache - MOH - or of probable MOH) was reported between 1.2% in India’s Karnataka State [23] and 6.1% in Brazil [24]. Among pediatric populations, prevalence of EM was lower, i.e. between 9.2% [29] and 10.6% [38].

### Overall impact of migraine disorders

Overall impact of EM and CM was the most common theme, as it was reported in 31 studies [13, 17, 21–23, 25, 26, 30, 32, 36, 37, 39–59]. In most of these studies, impact was addressed using disability measures, e.g. the MIDAS [11] or the Headache Impact Test (HIT-6) [66], and quality of life (QoL) measures, e.g. the 36-item Short Form Health Survey (SF-36) [67] or the Migraine-Specific Quality of Life Questionnaire (MSQ) [68]. Overall, results based on a MIDAS-like approach, i.e. on the division between days with limitations in work, household tasks and leisure time activity, show that most of limitations are referred to days with reduced household and leisure activities rather than to the work-related ones [17, 21, 22, 25, 43, 44, 46, 52–54]. All studies in which patients with migraine were compared to those with TTH, or to non-headache patients, showed that people suffering of migraine had worse disability or QoL, and that higher headache frequency was associated to worse health status [13, 17, 25, 26, 30, 43, 48, 50–53, 55, 58]. Less common topics include the impact of menses-related migraine, which is associated to higher disease burden [45], and to the impact of multimorbidity status: in this manuscript, patients with two or more comorbidities were shown to have a higher disability and lower QoL compared to those with one or no comorbidities [40].

### Impact on work or school activities

The impact of EM and CM on work or school activities was the second most frequent theme, and it was reported in 22 studies [14, 17, 21, 22, 25, 34, 37–41, 43, 47, 48, 54, 55, 58–62].

Two papers were on limitations in school-related activities [38, 48]: the first showed that 10.6% of school-aged children suffer from migraine and that they missed on average 4.1 school-days per year; the second paper reported that almost half of students suffering from migraine reduced or missed some school-days, and that their parents too missed some workdays to care for their children with migraine.

The majority of studies (20 out of 22) were on migraine impact on work productivity. In 15 studies, sufficient information to calculate three-month lost productive time, defined as one unit for each full workday lost and half unit for each day worked with reduced productivity, was available: on average patients with migraine lost between 3.2 and 89.2 work-equivalent days per year, on average 10.2 days. Most relevant reduction is due to presenteeism: in fact, on an annual basis, patients lost on average 4.4 workdays, but worked with reduced productivity for further 11.4 days.

In addition to these common issues, one paper showed that patients reporting higher difficulties in work-related tasks also show problems in tasks unrelated to work, thus addressing the issue of pervasiveness of migraine on different life domains [47]. Finally, a recent publication presented the validation of the HEADWORK questionnaire, a new instrument specifically developed to address limitations in work-related activities and the factors contributing to these difficulties [39]. HEADWORK questionnaire has good metric properties and the validation study showed that higher work-related difficulties are associated to headache frequency, pain intensity, perceived productivity reduction, female gender and CM status (vs. EM).

### Impact on family life

The impact of migraine disorders on family life was reported in five studies [17, 48, 54, 56, 63] with quite heterogeneous results. Family burden was in fact shown to increase consistently with increased migraine headaches frequency [17], and most of the limitations reported by patients were referred to caring for and dealing with their children [56]. Two papers focused on the burden of being a caregiver of a person with migraine: in the first it was shown that being a partner of a migraineur has moderate/strong impact on family life and leisure time [54]; in the second, it was shown that caregiving for children with migraine was associate to parents’ reduced workforce participation [48]. Finally, the last paper was on the development of the scale Impact of Migraine on Partners and Adolescent Children (IMPAC), which showed good metric properties and addresses the impact of migraine on family activities in general, on relationships with partners and on relationships with children [63].

### Interictal burden

Interictal burden was described in three studies [9, 17, 64], that reported complementary results. Interictal burden was in fact shown to increase consistently with increased migraine headaches frequency [17] and, in fact, it was reported by 10–26% of EM patients and by 29–41% of patients with probable MOH [9]. With regard to the “content” of the term interictal burden, the third paper showed that, during interictal phases, migraine patients spent relatively less time being active compared with healthy controls and, when active, their overall personal mobility level was lower and they also reported higher sleepiness and lower vigor [64].

### Disease costs

Finally, EM and CM costs were reported in eleven studies [27, 34, 35, 41, 44, 49, 59–62, 65] covering the period 2001–2017. Within these studies, several differences in the total cost were shown, which are likely due to the type of cost structure adopted in each study, i.e. focusing on direct costs rather than on both direct and indirect ones, as well as of the year in which the survey was carried out. In general, studies addressing both EM and CM show that the costs of CM are three to four-fold than those of EM. The most recent US-wide study shows that total cost of EM was 2649$/year, and that the cost of CM was 8243$/year: in this study, 60–64% of migraine costs was due to direct medical ones [40]. The most recent Europe-wide study showed that the average direct cost of EM was 746€/year, and that those of CM were 2427€/year [49]. The majority of these studies are based on population polls, but there are two recent exceptions to this, both referred to clinical samples of Italian patients. The first study addressed direct healthcare costs only and showed that direct healthcare cost of EM was 521€/year, while that of CM was 2250€/year [44]. In the second study, patients with CM and MOH were enrolled at the time-point of structured withdrawal in a headache center, i.e. whey they have the worst clinical situation and, probably, the highest costs: the estimated one-year CM costs were 10,730€, and approximately 39% of that cost (i.e. around 4185€) was indirect [59].

## Discussion

The fact that headache disorders, and migraine in particular, are burdensome conditions has been repeated so frequently in research papers to the point that such a kind of a statement is a sort of “starting point” in several papers. The results of this narrative review show that the meaning of a sentence like “migraine is a burdensome condition” is not univocal. Rather, there are at least six main themes that have been associated to the broad concept of burden and impact of migraine: prevalence of migraine, its overall impact (mostly defined as reduced QoL or disability), impact on work or school activities, impact on family life, interictal burden and disease costs.

The issue of high prevalence of migraine is of great epidemiological relevance and it is a “strength” when its burden has to be compared against other diseases. In fact, the 2010 version of the European Brain Council paper on “Cost of disorders of the brain in Europe” showed that headache disorders were the less costly and more prevalent conditions [15], and migraine, in terms of associated disability expressed with YLDs, was ranked at the second place after low back pain, being responsible of 5.6% of all YLDs, but it ranked first among the subgroup of people aged 50 years of less [69], i.e. the age group deeply involved into family and work duties.

It is therefore not casual if themes such as impact on work or school activities and impact on family life are of increasing interest. In fact, in the last decade the presence of such themes in available research on the impact of migraine is more than doubled, and condition-specific assessment instruments for detecting migraine impact on work and family life (i.e. the HEADWORK questionnaire [39] and the IMPAC scale [63]) have been developed in the last two years. These new assessment instruments have the potential to make a difference in the way in which these themes will be represented in future research, similarly to what happened for overall impact, which was the most reported theme. In fact, the three most commonly used assessment instruments for addressing disability and QoL, i.e. the MIDAS [11], the HIT-6 [66] and the MSQ [68] were published between 1999 and 2003, with the result of a relevant increase in the production of evidence connected to the theme “overall impact”.

The issue of interictal burden received less attention so far. We hypothesize that the reason for this lies in one of the core features of EM, i.e. its episodicity: in fact, by definition migraine headache attacks last 4–72 h, which might have led researchers to focus on the reduction of functioning during attacks. Conversely, in the case of CM, the interictal periods may be considered of lower relevance since patients spend the majority of time (i.e. 15 or more days per month) in ictal phases. In recent years, the importance of interictal phases in migraine has been increasingly recognized, mostly in basic-science covering vascular, neurophysiological, neuropsychological or neuroimaging aspects of migraine [70–74]. However, the fact that migraine may impact on patients’ lives also during interictal phases has been shown [9, 10], and the importance of being a migraine sufferer also during interictal phases is witnessed by some items of the MSQ questionnaire, such as item 1 (*How frequently have migraines interfered with how well you dealt with family, friends and others who are close to you*?) or item 9 (*How frequently did you need help in handling routine tasks such as every day household chores, doing necessary business, shopping, or caring for others, when you had a migraine*?). Despite this, the burden and impact of migraine during interictal phases is still neglected and deserves more research.

Migraine cost is a relevant and complex public health issue as it requires a broad knowledge of the impact of migraine not only on direct healthcare costs but also on indirect ones, i.e. those connected to reduced ability to work, as well as on other “intangible” aspects, such as the time spent searching for proper care or the time taken away from one’s own family duties. Since 1999, with the publication of the MIDAS as a disability measure [11], attention has been given to both lost workdays (absenteeism) and to days worked with reduced productivity due to migraine (presenteeism). Our analysis shows that the economic impact of migraine, a disease usually affecting people in the productive age, is mostly due to presenteeism rather than to absenteeism: in fact, the overall number of days with reduced productivity is approximately 2.6 fold the entire number of lost workdays (4.4 vs. 11.4), as reported in some of the studies herein included [34, 40, 59, 61, 65]. However, defining the total costs of migraine on the basis of the literature herein collated is problematic for three main reasons. First, studies have been published in different periods, and therefore the comparison between costs incurred in different time points of data collection and present costs is problematic. Second, different studies used different approaches to the definition of costs (i.e. relying on pre-defined and more or less precise cost categories for drugs, hospitalization, diagnostics and so on), and in some occasions focused on few aspects only: for example, some studies addressed only a portion of direct healthcare costs, such as drugs and diagnostic procedures [44, 49]. Finally, our specific search was not on migraine costs, so some relevant studies might have been excluded.

It has to be acknowledged that, in the majority of cases, the main results of the studies included in our narrative review can be summed up in few words. In general, patients with migraine reported an higher burden or impact, defined with one or more of the six main themes herein identified, compared to non-headache patients or to patients with TTH, with a tendency towards worse outcomes consistently with higher headache frequency. The themes we identified have been differently studied and reported throughout the years, and some may be of higher importance in the future: in particular, we believe that the two themes impact on work-related activities and impact on family life should be expanded in future research for the following reasons. First, prevalence data show that migraine mostly affects people of working age, and women in particular: therefore the two themes impact on the core of professional and personal life of most of migraine patients, with women in particular being at risk of carrying a “double burden”. Second, there is a direct connection between the impact of migraine on work-related tasks and reduced productivity, but the degree of such association is still to be verified. Disease-specific questionnaires for the evaluation of migraine impact on work-related tasks, like HEADWORK, might be used to produce reliable work-related disability weights in studies evaluating the burden of EM and CM. These weights could then be exploited for producing reliable estimates on the burden and costs of migraine. The issue of impact of migraine on employment (and vice-versa) is an open one due to the amount of information that is still needed to understand how to support people with migraine in the workplace. This is of importance in consideration of the changes in labor market, e.g. the increasing relevance of smart working and flexibility in time and places, which will give a different meaning to labor policies aimed to promote work maintenance of people with chronic conditions like migraine. Third, an indirect connection exists between the impact on family life, of patients as well as of their family members, and several other themes, such as interictal burden and reduced QoL but also disease costs. In fact, as also shown in some of the papers included in our narrative review, living with a migraine sufferer – either an adult or a child – might impact on work duties of caregivers and increases the burden due to caregiving activities [48, 54]. Caregiving for migraine patients is still a neglected issue but it can be easily understood that if a person, especially an adult, is unable, during and between headache attacks, to carry out daily household chores due to migraine, someone will have to take care of these activities. Such an aspect of migraine burden is considered as “intangible”, but it is actual to patients and their family members like other aspects are, and it could be expected that it is a driver of reduced QoL in patients and relationship satisfactions in partners.

Some limitations need to be acknowledged in the interpretation of our results. First, our review cannot be considered as a systematic one and does not purports to be systematic: we limited our scope to few selected keywords that we believed could be the most representative terms. It is clear that focusing much more on issues such as disability and QoL, or using some of the terms that we could reasonably expect to underline burden concept (e.g. the terms “interictal” or “prevalence”) within the search strategy, would have led to more studies. Such a procedure, however, would have implied a pre-definition of terms, thus contrasting the idea of looking for the way in which burden is conceptualized in migraine research. Future reviews, in which a systematic approach is employed, could be carried out to address the coverage or these themes in literature as well as the consistency of results. Second, although the corresponding authors were contacted by e-mail, two papers could not be found and, more in general, we cannot be sure that all relevant articles were included. Third, we relied on MedLine only for our search strategy, which has potentially hampered the scope of our narrative review. We made this choice with the aim of reducing the amount of records in which clinical descriptions of participants to studies are based on self-reported diagnoses: considering how common headache disorders are, the possibility that patients incorrectly self-identify themselves as having migraine instead of other headache is concrete.

## Conclusions

In conclusion, we performed a narrative literature review aimed to identify the most common topics underlying the concepts of burden and impact of migraine, and selected 49 papers covering 25 years. Six main themes were identified: prevalence of migraine disorders, overall impact of migraine disorders, impact on work or school activities, family impact, interictal burden, and disease costs. In general, results show that patients with EM or CM reported an higher burden or impact compared to non-headache patients or to patients with TTH, with a tendency towards worse outcomes that is consistent with headache frequency.

Future research should focus much more on impact on work-related activities and on family life, including the issue of caregiving. The main reasons lie in the following: a) the epidemiology of migraine, which mostly affects people in the period of family caring and professional achievements; b) the direct connection between impact on work-related tasks and reduced productivity, which has an impact on disease costs, and is of importance in consideration of “new” labor market features (e.g. short time contracts, and smart working), that will give a different meaning to labor policies aimed to improving inclusion, work ability and employability of people with migraine; c) the connection between impact on family life and several other themes, such as interictal burden, reduced QoL and disease costs. Specific assessment instruments for these topics, such as the HEADWORK questionnaire [39] and the IMPAC scale [63] have recently been developed and should be implemented in research to enhance our understanding of the migraine burden.

A better understanding of the issues behind migraine burden could translate into a change in the primary policy framework of interest for patients with migraine, namely the welfare and health systems. The first should provide support to this large amount of population by enhancing welfare policies, such as employee management, sick leave and time off compensation schemes. The health sector should instead deliver services that not only deal with proper diagnosis and care but also with occupational health, such as the creation of safe and healthier work environments.

## Abbreviations

CM: Chronic Migraine
DALYs: Disability-Adjusted Life Years
EM: Episodic Migraine
GBD: Global Burden of Disease
HIT-6: Six-item Headache Impact Test
IMPAC: Impact of Migraine on Partners and Adolescent Children scale
MIDAS: Migraine Disability Assessment
MOH: Medication Overuse Headache
MSQ: Migraine-Specific Quality of Life Questionnaire
QoL: Quality of Life
SF-36: 36-item Short Form Health Survey
TTH: Tension-Type Headache
YLDs: Years Lived with a Disability
